# Job vacancies at the European Centre for Disease Prevention and Control (ECDC)

**DOI:** 10.2807/1560-7917.ES.2021.26.11.2103182

**Published:** 2021-03-18

**Authors:** Eurosurveillance editorial team

**Affiliations:** 1 European Centre for Disease Prevention and Control (ECDC), Stockholm, Sweden

The European Centre for Disease Prevention and Control (ECDC) invites applications for the following positions:

 

**Policy Expert Communicable Diseases Prevention and Control**
**Expert Infectious Diseases Epidemiology**

The application deadline expires on 12 Apr 2021. 

 

**Expert Microbial Safety of Substances of Human Origin**

The application deadline expires on 26 Apr 2021. 

 

For more information, please visit the ECDC website: 

https://ecdc.europa.eu/en/work-us/vacancies?f%5B0%5D=deadline_date%3A1

